# Hyaluronic acid injections for chronic tennis elbow

**DOI:** 10.1186/s13102-022-00399-0

**Published:** 2022-01-12

**Authors:** Gershon Zinger, Alexander Bregman, Ori Safran, Shaul Beyth, Amos Peyser

**Affiliations:** 1 Department of Orthopedic Surgery, Shaare Zedek Medical Center and Faculty of Medicine, Hebrew University of Jerusalem, 12 Shmuel Bait Street, 9103102 Jerusalem, Israel; 2 Hadassah Medical Center, Kiryat Hadassah, POB 12000, 91120 Jerusalem, Israel

**Keywords:** Epicondylitis, Tennis elbow, Tendinosis, Hyaluronic acid

## Abstract

**Background:**

For most patients, tennis elbow (TE) resolves within 6 months of onset. For those with persistent and painful TE, nonsurgical treatment options are limited. Thousands of studies have tried to find effective treatments for TE but have usually failed. In this study, we tested the hypothesis that injections with hyaluronic acid (HA) would be effective at reducing pain from chronic TE.

**Methods:**

Patients with a minimum of six months of pain from TE and with a pain level of 50 or greater (out of 100) were included in the study. They were randomized equally into one of two treatment groups: injection with HA or injection with saline control. Follow-up was conducted at 3, 6 and 12 months from the initial injection. Both the patient and the examiner at the follow-up visits were blinded to the treatment arm. The primary outcome measure was the visual analog scale (VAS pain) score at one year. Additional outcome measures included the shortened Disabilities of the Arm, Shoulder and Hand questionnaire (QuickDASH) and Patient Rated Tennis Elbow Evaluation (PRTEE) scores.

**Results:**

Eighteen patients were randomized into the HA injection treatment arm, and 17 (94%) completed the study. The average age was 51.9 years, and 10 of the subjects were male. Patients had an average of 28.1 months of pain before entering the study. The VAS score in the HA group decreased from a baseline of 76.4–14.3 at 12 months. All 17 patients in the HA group showed VAS score reductions above the minimal clinically important difference (MCID) of at least 18. The PRTEE score improved from 67 to 28.1. The QuickDASH score improved from 53.7 to 22.5. Follow-up in the saline group was less than 50% and was therefore not used as a comparator.

**Conclusions:**

HA injections yielded significant success in pain relief by three months. Patients continued to improve for the 12-month duration of the study. This study indicates that patients with chronic lateral epicondylitis may benefit from receiving injections of hyaluronic acid rather than having to undergo surgery.

## Background

Tennis elbow (TE) is a widespread and painful condition. Although usually a self-limited condition, in approximately 20% of cases, the pain remains chronic (ref). Nirschl [1], in his classic description (ref), describes angiofibroblastic dysplasia as a condition occurring primarily at the extensor carpi brevis (ECRB) origin that results from tendon microtears with a local avascular environment that prevents healing. Traditional nonoperative treatment for TE often starts with physical therapy and nonsteroidal anti-inflammatory medication. However, these treatments have not proven effective [2]. Local treatment commonly includes injection with steroids which, in double-blinded controlled studies, have been shown to give only temporary relief [3–5]. Other less common substrates for injection have included autologous blood, platelet-rich plasma (PRP) and Botulinum, none of which have proven effective. Autologous blood has limited evidence in the literature [6, 7]. Botulinum has shown a partial benefit that is only temporary and has the potential side effect of paresis [8]. PRP has been tried for over ten years with limited success; a recent review has actually recommended against using PRP as a treatment for TE. [9].

Tennis elbow is considered to be self-limiting; in 80% of patients, the symptoms resolve within six to twelve months [10]. However, for individuals with persistent and painful TE, the data supporting successful nonoperative options are limited. Recent studies have evaluated the injection of hyaluronic acid (HA) for tendinosis [11–14], specifically for tennis elbow [15, 16], and have shown promising results. Dong et al. [17], in a comprehensive review of injection therapy for tennis elbow, searched 1,636 titles and reviewed 27 randomized controlled trials (RCTs) that met their criteria. With regard to pain scores, hyaluronate injections were superior to all other treatments, but the researchers noted that more study was needed. Most of the studies done to date using HA for enthesopathies have included different enthesopathies in the same investigation and have performed limited follow-up [11–15]. One exception is a published level 1 study in which HA injections for tennis elbows showed promising results [16]. However, this study was limited to patients who were racquet sport athletes, although tennis players account for only 5–8% of patients who present with TE [16]. Therefore, the purpose of the current study was to expand the population to see if HA is effective in the general population not limited to competitive racquet sport athletes. Here, we prospectively evaluated the efficacy of HA injections for the treatment of chronic tennis elbow.

## Methods

This study was designed according to the Consolidated Standards of Reporting Trials (CONSORT) guidelines [18]. This clinical trial was prospective, randomized and blinded. The trial was registered at ClinicalTrials.gov (NCT02258295) before IRB approval. After meeting the inclusion and exclusion criteria, patients were randomized in a 1:1 ratio into one of two treatment arms: HA injection (HA group) versus saline control (saline group). All patients were recruited and evaluated at a single center, an academic referral facility. After providing informed consent, patients were randomized to HA versus saline injection in a 1:1 ratio. Randomization was performed using a random number generator provided by our statistician.

### Inclusion/exclusion criteria

The criteria for diagnosis included pain and tenderness at the lateral epicondyle that worsened with resisted wrist or finger extension (with the elbow in the extended position). The inclusion criteria were age over 18 years, chronic pain defined as six months or longer, and pain (average pain over the past week when using the hand) measured on the visual analog scale (VAS) at 50 mm or greater (out of 100 mm).

Exclusion criteria included elbow steroid injection less than three months prior to starting the study, prior elbow surgery, inflammatory conditions such as rheumatoid arthritis or lupus, and allergies to birds, feathers or egg products. If the patient had complaints of pain and significant tenderness on exam in the area of the radial neck, then a component of radial tunnel syndrome was assumed, and those patients were excluded from the study. Patients with pain from other areas, such as the radiocapitellar joint or medial epicondyle, were also excluded.

### Blinding

All injections were performed using syringes that were masked and numbered. The patients were blinded to the treatment arm. The return evaluations at 3, 6 and 12 months were performed by an experienced hand specialist who was also blinded to the treatment arm.

### Injections

This study used Intragel (IBSA Institut Biochimique, Lugano, Switzerland). The formulation has a molecular weight averaging 800–1200 kDaltons and a concentration of 16 mg per 2 cc. The author (GZ) performed all injections. The injections were performed in a similar fashion for both the HA and saline groups. First, the point of maximum tenderness at the lateral epicondyle was identified and marked. After local preparation with alcohol, 1 cc of lidocaine 1% was placed both superficially and deep into the tendon substance. Using a separate and preloaded syringe, 2 cc of either HA or saline was injected using a fanning technique of 3 perforations into the area of maximal tenderness approximately 1 cm distal to the lateral epicondyle. Each participant was injected three times, two weeks apart. When planning the study, we reviewed all the clinical studies to date, and the number of injections performed was between one and six per patient.

### Additional treatment

Patients were not referred for any additional treatment during the study period. Most had tried therapy and steroid injections prior to enrollment.

### Demographic data

General demographic data included age, sex, handedness, type of work, symptomatic side, and participation in racquet sports (Table 1).

**Table 1:** Categorical (Chi square test for gender, Fishers test for others—for comparisons between groups) and continuous (T-tests and Wilcoxon for normal and non-normal distributions) baseline characteristics by group: HA versus saline

Parameter	Category	Group HA N (%)	Group saline N (%)	P-value
Age (years)*	Age	51.9 (SD 10.6)	52.9 (SD 8.9)	0.800
Gender	Female	7 /17 (41.2)	3 /13 (23.1)	0.297
	Male	10 /17 (58.8)	10 /13 (76.9)	
Handedness	Left	1 /17 (5.9)	1 /14 (7.1)	1.00
	Right	16 /17 (94.1)	13 /14 (92.9)	
Occupation	Manual	3 /17 (17.6)	4 /14 (28.6)	0.115
	Office	13 /17 (76.5)	6 /14 (42.9)	
	Retired	1 /17 (5.9)	4 /14 (28.6)	
BMI	BMI	25.9 (SD 3.2)	27.1 (SD 4.1)	0.463
Painful side	Left	8 /16 (50.0)	7 /14 (50.0)	1.00
	Right	8 /16 (50.0)	7 /14 (50.0)	
Pain Duration (months)	Pain Duration	28.1 (SD 22.0)	51.4 (SD 59.9)	0.936
VAS pain (in the past week how much pain do you feel when gripping something—on average?)	VAS pain	76.4 (SD 12.1)	72.1 (SD 11.9)	0.348
PRTEE Score	PRTEE	67.0 (SD 14.6)	71.9 (SD 14.5)	0.357
QuickDASH	QuickDASH	53.7 (SD 18.90	58.8 (SD 13.1)	0.408
Racquet sports	No	15 /17 (88.2)	13 /14 (92.9)	1.00
	Yes	2 /17 (11.8)	1 /14 (7.1)	

HA—Hyaluronic Acid, PRTEE—patient-rated tennis elbow evaluation, QuickDASH—Quick Disabilities of the Arm, Shoulder and Hand Score, VAS—Visual Analog Score

*T test

### Outcome measures

The primary outcome measure was the VAS score for pain when the subject was asked, “What is the average pain you experienced the past week while gripping or actively using your hand?” Secondary outcome measures included the brief form of the Disabilities of the Arm, Shoulder, and Hand questionnaire (QuickDASH) [19] and the Patient-Rated Tennis Elbow Evaluation (PRTEE) [20]. Outcome measures were collected at baseline, three months, six months and one year from the initial injection. Patients were encouraged to return for clinical evaluation for each visit, but some preferred to respond to outcome questionnaires by telephone or email.

The QuickDASH is an 11-question short version of the longer 30-question DASH. The score ranges from 0 (no disability) to 100 (most severe disability). The PRTEE is a 15-question survey that evaluates pain and function on a 10-point VAS. The score ranges from 0 (no pain and maximum function) to 100 (maximum pain and minimum function).

### Primary endpoint

The primary endpoint was a reduction in VAS pain three months after the initial injection.

### Secondary endpoints

Secondary outcomes included differences in HA treatment outcomes in terms of VAS pain at six and 12 months and for PRTEE and QuickDASH at 3, 6 and 12 months post-injection. We also calculated a 25% reduction in VAS pain from baseline for HA versus saline to allow comparison to the Peerbooms et al. 2010 [21] results from PRP injection.

### Strength of study and statistical analysis

One of the few prospective studies on HA performed to date was conducted by Petrella et al. [16]^.^ They evaluated the treatment of chronic TE in racquet sport athletes using a total of two HA injections one week apart. They used pain VAS scores as their primary endpoint. Using standard deviation data from their study, we calculated the sample size needed to power this study. With the null hypothesis that the HA group would improve relative to the control group (by VAS 18 or greater) at 3 months post-injection, the significance level, $\propto$ set at 0.05 and power at 80% (1-$\beta$) = 0.20, computed 29 patients per group to allow comparison to the saline placebo. Unfortunately, the inclusion and exclusion criteria were so restrictive that enrollment was slower than anticipated. Specifically, the requirement for only chronic TE pain, no component of radial tunnel syndrome and no recent steroid injections limited the number of suitable patients. In the end, we stopped the study enrollment at 35 patients, with 18 in the HA group.

Differences in baseline characteristics were assessed with Fisher’s test for categorical variables and the T test or Wilcoxon test for continuous variables, depending on the distribution of the data. The T test was used to evaluate differences in outcome measures.

## Results

**Fig. 1:**
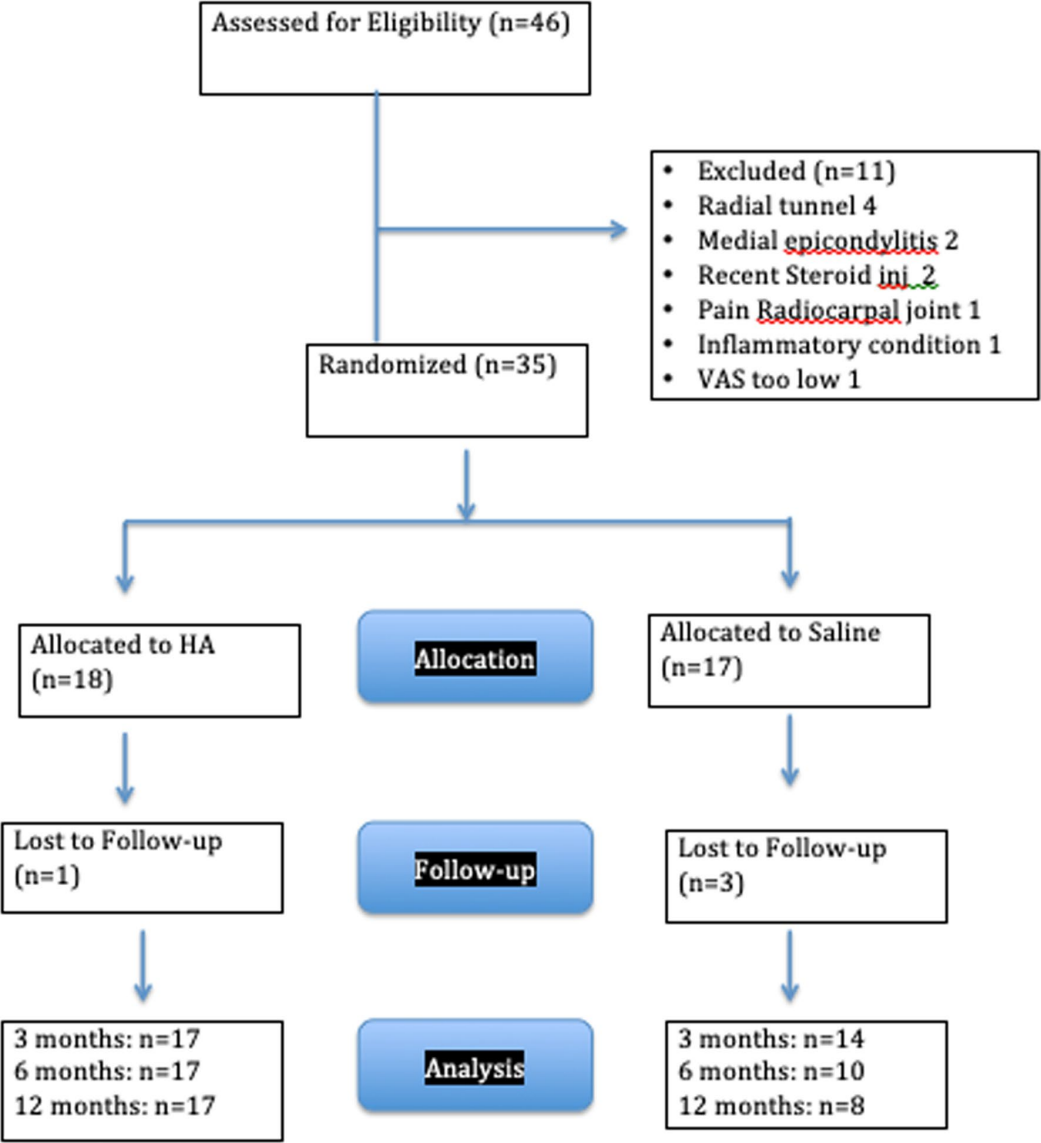
Flow diagram

The enrollment period was from January 18, 2017, to December 3, 2018, and the study period continued for one year from the final injection until December 2019. In the saline group, 17 patients were initially enrolled. There was increasing drop-out at each follow-up visit so that by the 12-month visit, only eight of the 17 patients (47%) returned for follow-up (Fig. 1). Although we attempted to contact these patients, we were not able to reach them to have them return for follow-up evaluation. Since we could not analyze the information from the saline-treated patients, we did not include their information in the analysis. Therefore, this study should be considered a prospective study describing the effects of HA injections for chronic TE patients.

In contrast to the saline-injected group, of the 18 patients enrolled in the HA group, 17 returned for follow-up appointments for the full year of the study. The single patient lost in the HA group did not return for their first 3-month follow-up and was not counted in the outcome measures.

Demographic data were collected at the initial visit after randomization and were equivalent (Table 1). Although six months was the minimum duration of pain to be included in the study, the average pain duration was 28.1 (SD 22) months. No complications, including subcutaneous atrophy, infection, or pain flare from the injection, were noted in any of the patients in the study.

### Primary outcome

The VAS pain score improved in the HA group from a baseline of 76.4 (SD 12.1) to 42.6 (SD 25.5) at three months (p = 0.001).

### Secondary outcomes

#### Pain measures (using last carry forward)

**Fig. 2:**
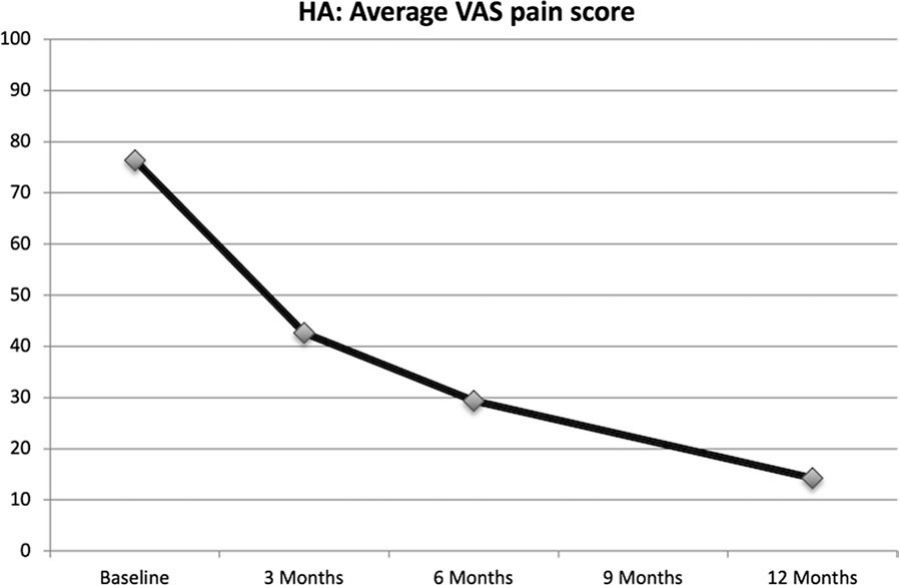
Average VAS pain levels

The average pain score in the HA group continued to improve over time (Fig. 2). The average VAS score improved 12 months after treatment in the HA group, from 76.4 (SD 12.1) to 14.3 (SD 11.9) (p < 0.001).

#### Additional VAS pain reduction measures

**MCID (minimal clinically important difference):** All 17 patients in the HA group showed VAS score improvement above an MCID of at least 18 [22].^.^

**Twenty-five percent reduction:** Using Peerbooms et al. 2010 [21] criteria of 25% or more improvement, when evaluated at 12 months, all 17 patients in the HA group met that criterion.

**Fig. 3:**
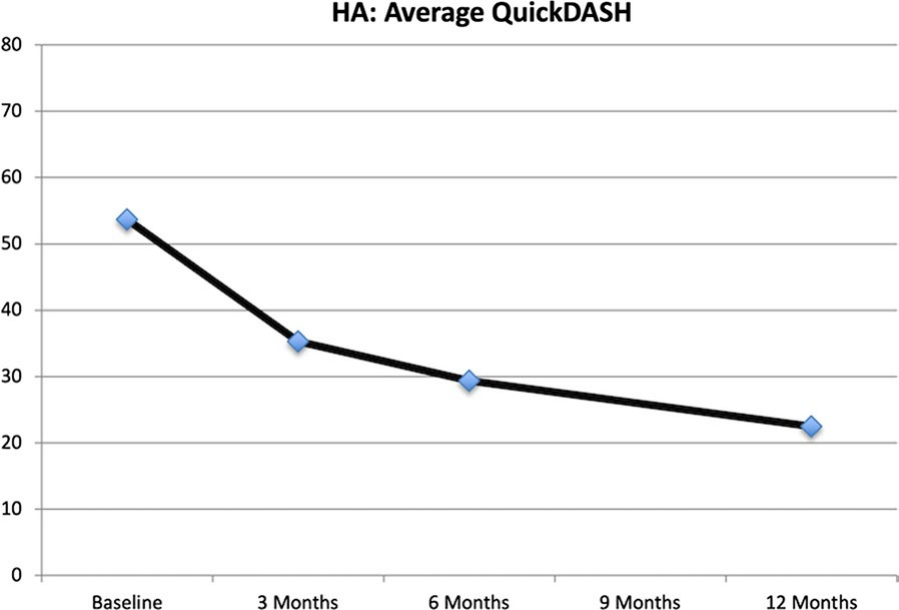
Average QuickDASH scores

**QuickDASH:** The QuickDASH score improved over time (Fig. 3), in the HA group, from 53.7 (SD 18.9) to 22.5 (SD 17.1) (p < 0.001) at 12 months. This average difference of 31.2 is above the MCID of 14. [23].

**Fig. 4:**
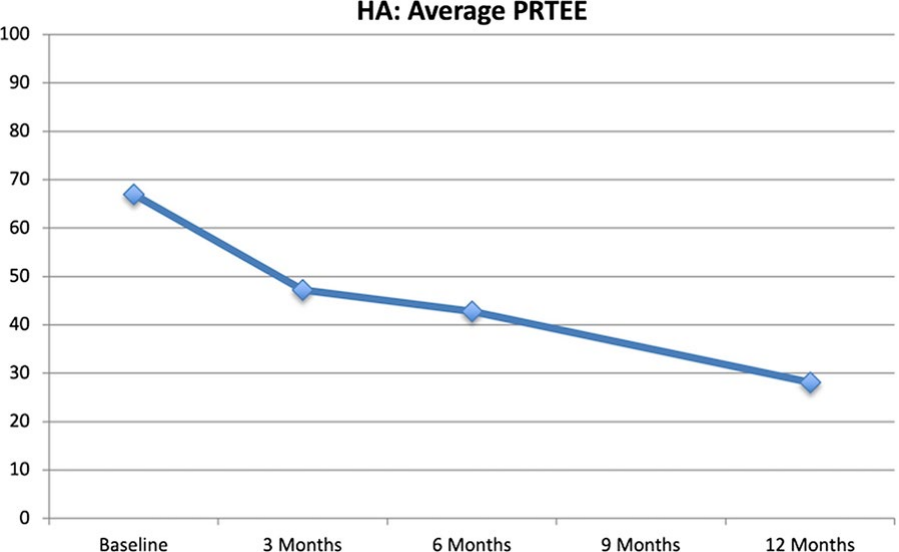
Average PRTEE combined (pain and function) scores

**PRTEE:** The PRTEE score improved over time (Fig. 4). The HA group improved from 67.0 (SD 14.6) to 28.1 (SD 15.8) at 12 months (p < 0.001). Poltawski et al. [24] evaluated the MCID for the PRTEE and reported that 37% improvement correlated with “much better” or “completely recovered”. In the HA group, 14 of the 17 patients met this criterion.

Both the QuickDASH and the PRTEE measure pain and function. The PRTEE is considered a more specific measure for tennis elbow and theoretically would be more sensitive to changes when evaluating patients limited by TE. In this case, both measures improved since patients improved in both groups with less pain and more function.

## Discussion

The results of this prospective study show that HA injections were effective at relieving pain and improving function in patients with chronic TE. Despite an average of more than two years of pain, the VAS score improved from 76.4 to 14.3.

A patient with chronic tennis elbow has few proven options other than surgery. Coombes et al. [3] performed a systematic review using eight databases and identified 3,824 trials of peritendinous injections for tendinopathy. Forty-one studies met their inclusion criteria. Other than injections of sodium hyaluronate, there was no intervention that gave more than temporary relief. Crimaldi et al. [25] reviewed HA for use in tendinopathies in a recent publication. They cited preclinical studies with a mechanism of action that included reduction of proinflammatory markers, improved tenocyte viability and tendon repair. They reviewed clinical studies that demonstrated benefits in upper and lower extremity sport-related tendinopathies and concluded that further research is needed.

Steroid injections continue to be the most common treatment, and PRP has become popular despite insufficient scientific support. In a prospective, double blind randomized clinical trial of 64 patients with less than six months of pain, Lindenhovius et al. [4] concluded that steroid injection did not affect the self-limited course of lateral elbow pain. Most of the literature on PRP contains case reports or case series [3, 26, 27]. One exception is the study by Peerbooms et al. [21], who reported their results from a randomized double-blinded study comparing PRP to steroid injection with a one-year follow-up. They defined successful treatment as 25% or better improvement in VAS scores compared to baseline, and they calculated 73% success in the PRP group versus the 100% found in this study using HA.

In a review of the English-language literature, we found eleven relevant studies that evaluated HA for tendinopathies. Three used HA for lateral epicondylitis [15, 16, 28]; three, for the rotator cuff; [11–13] one, for the Achilles [14]; and one was an animal study [8]. Three studies evaluated HA injection for multiple tendinopathies [29–31]. All the studies described here showed some benefit from HA injection but were of varying quality and did not limit the treatment to chronic tennis elbow; furthermore, most had only a short-term follow-up. The study by Gaughan et al. [32] offers an understanding of the pathomechanism for HA improvement. Horses had a flexor tendon defect created when injected with HA compared to methylcellulose, with the contralateral limb serving as a control. After killing the animals eight weeks after the injection, the researchers found histological evidence of HA-treated limbs with reduced inflammatory cells, improved tendon structure and fewer adhesions.

Petrella et al. [16] performed a blinded prospective randomized clinical trial of hyaluronate versus saline injection. They included 331 racquet sport athletes with chronic (> 3 months) lateral epicondylitis and measured VAS pain in addition to four other outcome measures. The results showed improved pain with grip in the HA-treated group, with VAS scores that improved from a baseline of 9.8 to 2.9 at one year.

### Saline control

The saline group was not compared to the HA group since it was considered an unreliable comparator. We tried to contact the lost patients and offer them treatment with HA or at least determine why they did not return, but they would not respond to either phone or email contact, which we purposely limited to two efforts each. We can only speculate as to the reasons for the high saline drop-out compared to the low-drop out for the successfully treated HA group.

There is some evidence that saline for TE may not be a true placebo but might also have therapeutic benefits. It therefore might not be the ideal comparator. Gao et al. [33] and Acosta-Olivo et al. [22] performed meta-analyses of the effect of saline injection for tennis elbow. They evaluated only prospective, randomized studies that had a minimum follow-up of a year. They concluded that the improvement seen with saline injection is not a placebo effect but rather that saline injections provide real therapeutic benefit.

### Surgery

Although the focus here is to compare HA injection to other nonsurgical treatments, it is worth comparing the results here to surgical treatment. Ruch et al. [34] compared preoperative to postoperative open treatment of tennis elbow after failed conservative treatment. The average VAS pain score improved from 4.6 to 2.3. Pierce et al. [35] performed a recent systematic review of open, arthroscopic and percutaneous techniques. They noted that the VAS pain score at the final follow-up was 1.9, 1.4 and 1.3, respectively. These findings compare to our results from HA injection, which yielded a VAS pain score that improved from 7.6 to 1.4.

### Merits and limitations

There are no studies to date that report the results of a prospective study of HA treatment for LE in non-racquet sport athletes with a follow-up of one year. The only other prospective study on HA treatment was performed by Patrella on racquet sport athletes [16]. This study also adds to the literature because of the variability in the different HA formulations and the number and frequency of the injections. Petrella et al. [16] did not specify the specific HA formulation they used and noted two injections a week apart. In this study, we used Intragel with a specific molecular weight and given concentration for a total of 3 injections 2 weeks apart. This information may be useful for designing future research. Another strength was the blinding of the patient and the evaluator.

The many patients lost to follow-up in the saline group limited this study to having a placebo group for comparison. However, as noted, saline may have some therapeutic benefits of uncertain duration and may not be the ideal placebo control. In addition, given our strict inclusion and exclusion criteria, there was lower than targeted patient recruitment. However, the HA group showed significant improvement using all measures.

## Conclusions

We conclude that, based on this prospective study with one year of follow-up, HA proved effective at treating chronic TE. Other than the pain of injection, no negative side effects of HA injection were observed over the course of the study. We feel that despite the limitations of this study, there is a large benefit and minimal risk that favors injecting HA for chronic tennis elbow. However, we recommend that a larger study with appropriate placebo control be performed. Tennis elbow and other enthesopathies remain difficult to treat. We hope this study stimulates further research in this important area to investigate the use of HA injections to treat this painful condition.

## Data Availability

The datasets used and/or analyzed during the current study are available from the corresponding author on reasonable request.

## Abbreviations

CONSORT: Consolidated Standards of Reporting Trials
HA: Hyaluronic acid
IRB: Institutional review board
QuickDASH: Shortened Disabilities of the Arm, Shoulder and Hand questionnaire
MCID: Minimal clinically important difference
PRP: Platelet-rich plasma
PRTEE: Patient-rated Tennis Elbow Evaluation
TE: Tennis elbow
VAS: Visual analog scale

## References

[1] Nirschl RP (1986). Soft-tissue injuries about the elbow. Clin Sports Med.

[2] Smidt N, Assendelft WJ, Arola H, Malmivaara A (2003). Effectiveness of physiotherapy, for lateral epicondylitis: a systematic review. Ann Med.

[3] Coombes BK, Bisset L, Vicenzino B (2010). Efficacy and safety of corticosteroid injections and other injections for management of tendinopathy: a systematic review of randomised controlled trials. Lancet.

[4] Lindenhovius A, Henket M, Gilligan BP, Lozano-Calderon S, Jupiter JB, Ring D (2008). Injection of dexamethasone versus placebo for lateral elbow pain: a prospective, double-blind, randomized clinical trial. J Hand Surg Am.

[5] Olaussen M, Holmedal Ø, Lindbæk M, Brage S (2009). Physiotherapy alone or in combination with corticosteroid injection for acute lateral epicondylitis in general practice: a protocol for a randomised, placebo-controlled study. BMC Musculoskelet Disord.

[6] Creaney L, Wallace A, Curtis M, Connell D (2011). Growth factor-based therapies provide additional benefit beyond physical therapy in resistant elbow tendinopathy: a prospective, single-blind, randomised trial of autologous blood injections versus platelet-rich plasma injections. Br J Sports Med.

[7] Kazemi M, Azma K, Tavana B, Rezaiee M, Farid PA (2010). Autologous blood versus corticosteroid local injection in the short-term treatment of lateral elbow tendinopathy: a randomized clinical trial of efficacy. Am J Phys Med Rehabil.

[8] Wong SM, Hui ACF, Tong PY, Poon DWF, Yu E, Wong LKS (2005). Treatment of Lateral Epicondylitis with Botulinum Toxin: a randomized, double-blind, placebo-controlled trial. Ann Intern Med.

[9] De Vos RJ, Windt J, Weir A (2014). Strong evidence against platelet-rich plasma injections for chronic lateral epicondylar tendinopathy: a systematic review. Br J Sports Med.

[10] Sayegh ET, Strauch RJ (2015). Does nonsurgical treatment improve longitudinal outcomes of lateral epicondylitis over no treatment? A meta-analysis. Clin Orthop Relat Res.

[11] Melon F, Milia F, Cavazzuti M (2008). Clinical evaluation of sodium hyaluronate in the treatment of patients with sopraspinatus tendinosis under echographic guide: experimental study of periarticular injections. Eur J Radiol.

[12] Sengül I, Öz B, Yolerl Ö, Ölmez N, Memiş A, Uluç E (2008). Sodium hyaluronate injections compared to local modalities for the treatment of shoulder impingement syndrome. Turk J Phys Med Rehab.

[13] Shibata Y, Midorikawa K, Emoto G, Naito M (2001). Clinical evaluation of sodium hyaluronate for the treatment of patients with rotator cuff tear. J Shoulder Elb Surg.

[14] Sundqvist H, Forsskh B, Kvist M (1987). A promising novel therapy for achilles peritendinitis. Double-blind comparison of glycosaminoglycan polysulfate and high-dose indomethacin. Int J Sports Med.

[15] Åkermark C, Crone H, Elsasser U, Forsskåhl B (1995). Glycosaminoglycan polysulfate injections in lateral humeral epicondylalgia: a placebo-controlled double-blind trial. Int J Sports Med.

[16] Petrella RJ, Cogliano A, Decaria J, Mohamed N, Lee R (2010). Management of tennis elbow with sodium hyaluronate periarticular injections. Sport Med Arthrosc Rehabil Ther Technol.

[17] Dong W, Goost H, Lin XB (2016). Injection therapies for lateral epicondylalgia: a systematic review and Bayesian network meta-analysis. Br J Sports Med.

[18] Rennie D (2001). CONSORT revised—improving the reporting of randomized trials. JAMA.

[19] Gummesson C, Ward MM, Atroshi I (2006). The shortened disabilities of the arm, shoulder and hand questionnaire (QuickDASH): validity and reliability based on responses within the full-length DASH. BMC Musculoskelet Disord.

[20] MacDermid J (2005). Update: the patient-rated forearm evaluation questionnaire is now the patient-rated tennis elbow evaluation. J Hand Ther.

[21] Peerbooms JC, Sluimer J, Bruijn DJ, Gosens T (2010). Positive effect of an autologous platelet concentrate in lateral epicondylitis in a double-blind randomized controlled trial: platelet-rich plasma versus corticosteroid injection with a 1-year follow-up. Am J Sports Med.

[22] Acosta-Olivo CA, Millán-Alanís JM, Simental-Mendía LE (2020). Effect of normal saline injections on lateral epicondylitis symptoms: a systematic review and meta-analysis of randomized clinical trials. Am J Sports Med.

[23] Sorensen AA, Howard D, Tan WH, Ketchersid J, Calfee RP (2013). Minimal clinically important differences of 3 patient-rated outcomes instruments. J Hand Surg Am.

[24] Poltawski L, Watson T (2011). Measuring clinically important change with the patient-rated tennis elbow evaluation. Hand Ther.

[25] Crimaldi S, Liguori S, Tamburrino P, Moretti A, Paoletta M, Toro G, Iolascon G (2021). The role of hyaluronic acid in sport-related tendinopathies: a narrative review. Medicina (Kaunas).

[26] Mishra A, Pavelko T (2006). Treatment of chronic elbow tendinosis with buffered platelet-rich plasma. Am J Sports Med.

[27] Mishra AK, Skrepnik NV, Edwards SG (2014). Efficacy of platelet-rich plasma for chronic tennis elbow: a double-blind, prospective, multicenter, randomized controlled trial of 230 patients. Am J Sports Med.

[28] Khan IU, Awan AS, Khan AS, Marwat I (2018). Efficacy of a single-injection sodium hyaluronate treatment in lateral epicondylitis. J Ayub Med Coll Abbottabad.

[29] Fogli M, Giordan N, Mazzoni G (2017). Efficacy and safety of hyaluronic acid (500–730kDa) ultrasound-guided injections on painful tendinopathies: a prospective, open label, clinical study. Muscles Ligaments Tendons J.

[30] Kumai T, Muneta T, Tsuchiya A (2014). The short-term effect after a single injection of high-molecular-weight hyaluronic acid in patients with enthesopathies (lateral epicondylitis, patellar tendinopathy, insertional Achilles tendinopathy, and plantar fasciitis): a preliminary study. J Orthop Sci.

[31] Lynen N (2012). Treatment of chronic tendinopathies with peritendinous hyaluronan injections under sonographic guidance—an interventional, prospective, single-arm, multicenter study, Dtsch. Arzte-Verlag.

[32] Gaughan EM, Nixon AJ, Krook LP (1991). Effects of sodium hyaluronate on tendon healing and formation in horses. Am J Vet Res.

[33] Gao B, Dwivedi S, DeFroda S (2019). The therapeutic benefits of saline solution injection for lateral epicondylitis: a meta-analysis of randomized controlled trials comparing saline injections with nonsurgical injection therapies. Arthroscopy.

[34] Ruch DS, Orr SB, Richard MJ, Leversedge FJ, Mithani SK, Laino DK (2015). A comparison of débridement with and without anconeus muscle flap for treatment of refractory lateral epicondylitis. J Shoulder Elbow Surg.

[35] Pierce TP, Issa K, Gilbert BT (2017). A systematic review of tennis elbow surgery: open versus arthroscopic versus percutaneous release of the common extensor origin. Arthroscopy.

